# Chitosan: An Update on Potential Biomedical and Pharmaceutical Applications

**DOI:** 10.3390/md13085156

**Published:** 2015-08-14

**Authors:** Randy Chi Fai Cheung, Tzi Bun Ng, Jack Ho Wong, Wai Yee Chan

**Affiliations:** School of Biomedical Sciences, Faculty of Medicine, The Chinese University of Hong Kong, Hong Kong, China; E-Mails: jack1993@yahoo.com (J.H.W.); chanwy@cuhk.edu.hk (W.Y.C.)

**Keywords:** chitosan, pharmaceutical applications, biomedical applications

## Abstract

Chitosan is a natural polycationic linear polysaccharide derived from chitin. The low solubility of chitosan in neutral and alkaline solution limits its application. Nevertheless, chemical modification into composites or hydrogels brings to it new functional properties for different applications. Chitosans are recognized as versatile biomaterials because of their non-toxicity, low allergenicity, biocompatibility and biodegradability. This review presents the recent research, trends and prospects in chitosan. Some special pharmaceutical and biomedical applications are also highlighted.

## 1. Introduction

Chitosan, sometimes known as deacetylated chitin, is a natural polycationic linear polysaccharide derived from partial deacetylation of chitin [1]. Chitin is the structural element in the exoskeleton of insects, crustaceans (mainly shrimps and crabs) and cell walls of fungi, and the second most abundant natural polysaccharide after cellulose. The complexity of the chitin structure, difficulty in its extraction and insolubility in aqueous solution limited the research on this polymer until 1980s.

**Figure 1:**
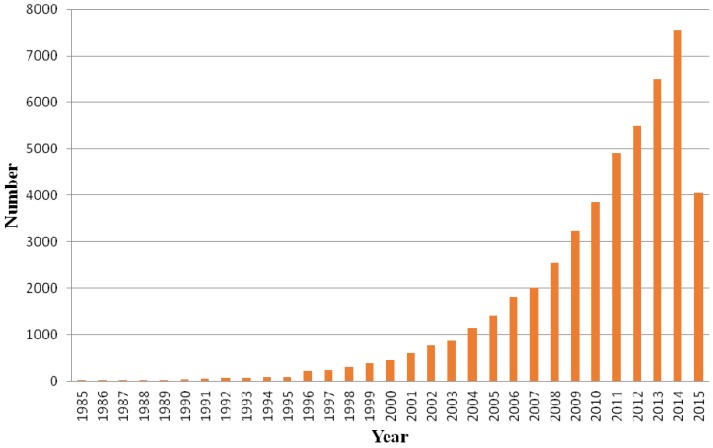
Scopus indexed publications related to chitosan and its derivatives.

Chitosan is composed of β-(1-4)-linked d-glucosamine and *N*-acetyl-d-glucosamine randomly distributed within the polymer. The cationic nature of chitosan is rather special, as the majority of polysaccharides are usually either neutral or negatively charged in an acidic environment. This property allows it to form electrostatic complexes or multilayer structures with other negatively charged synthetic or natural polymers [2]. The interesting characteristics of chitosan such as biocompatibility, non-toxicity, low allergenicity and biodegradability allow it to be used in various applications [3]. Besides, chitosan is reported to have other biological properties, such as antitumor [4], antimicrobial [5], and antioxidant [6] activities. The degree of deacetylation, which is described by the molar fraction of deacetylated units or percentage of deacetylation, and the molecular weight of chitosan, were found to affect these properties [7]. Chitosan has been widely used for different biological and biomedical applications recently due to its unique properties. For instance, it can be used in water treatment [8], wound-healing materials [1], pharmaceutical excipient or drug carrier [9], obesity treatment [10] and as a scaffold for tissue engineering [11]. There is increased interest in pharmaceutical as well as biomedical applications of chitosan and its derivatives and significant development has been achieved. It can be reflected in the increasing number of related publications throughout the years. Figure 1 shows the number of Scopus indexed publications from 1985 to June 2015 related to chitosan and its derivatives.

## 2. Production and Characterization of Chitosan

**Figure 2:**
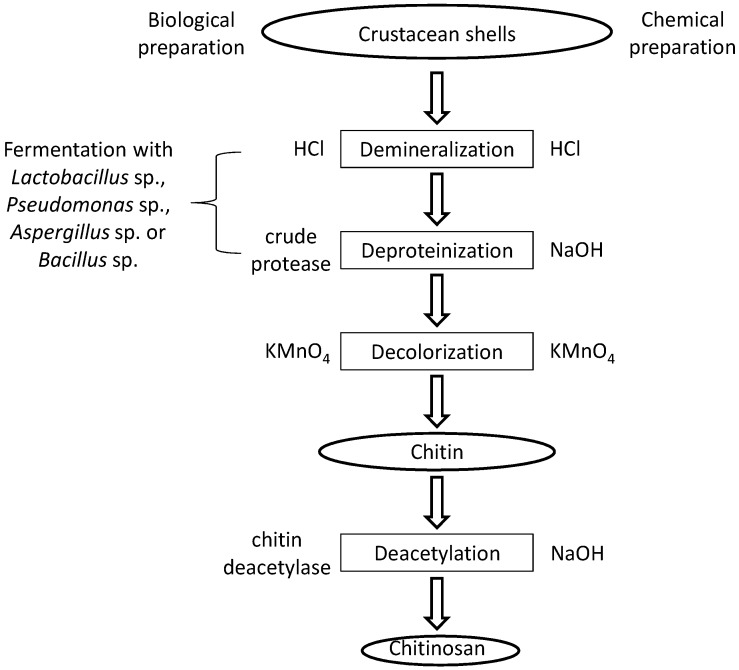
A schematic presentation of chitosan preparation from raw materials.

The raw material for the production of chitosan is chitin. The main sources are the shells of crustaceans, mainly crabs and shrimps. The purification process is easier for shrimp shells which are thinner. Usually, shells of the same size and species are grouped, then cleaned, dried and ground into small shell pieces [12]. There is no standard purification method as different chitin sources require different treatments due to the diversity in their structures. Conventionally, the protocol is divided into demineralization, deproteinization and decolorization steps which can be carried out using chemical [13] or biological (enzymatic treatment or fermentation) [14] treatments. The end-products need to be highly purified if they are to be used for biomedical or pharmaceutical purposes, as residual proteins, minerals or pigments can cause serious side effects. Conversion of chitin to chitosan can be achieved by enzymatic [15] or chemical [16] deacetylation. Chemical deacetylation is more commonly used for commercial preparation because of economic issues and feasibility for mass production. No matter which method is used, depolymerization is inevitable [17]. The processes involved in chemical and biological preparation of chitosan from crustacean shells are illustrated in Figure 2. 

The quality of chitosan depends on the source of chitin and its method of isolation [18] and chitosan with different extents of deacetylation are commercially available today. The applications of the chitosan depend on the characteristics, such as appearance of polymer, turbidity of polymer solution, degree of deacetylation and molecular weight [19]. The degree of deacetylation can be determined by different techniques, such as infrared spectroscopy, potentiometric titration, and more advanced methods like ^1^H liquid state and solid state ^13^C-NMR. The average molecular weight of chitosan is usually obtained from steric exclusion chromatography equipped with a viscometer and light scattering detector or matrix-assisted laser desorption/ionization-mass spectrometer [12]. Different characterization techniques for determining molecular weight, degree of deacetylation and crystallinity are summarized in Table 1. Chitosan obtained from deacetylation of chitin becomes soluble in aqueous acidic solutions when the average degree of deacetylation is above 0.5, but not at an alkaline or physiological pH. The physical properties of chitosan in aqueous solution depend on the degree of deacetylation and the acetyl group distribution in the polymer chains. Uneven acetyl group distribution will lower its solubility and make them form aggregates easily [20]. The solubility problem hinders its applicability. Modification of chitosan at the molecular level increases its solubility and stability and thus makes it more versatile as a biopolymer. The presence of free amino groups on the chitosan chains allows modifications under mild conditions. Chitosan usually reacts with other small molecules or polymers and is transformed into derivatives or composites. Chitosan hydrogel is one of the various forms of its composites. It is composed of a cross-linked network of polymer chains with a high content of hydrophilic groups. Thus, it is a superabsorbent of water, but is water-insoluble because of the chemical or physical bonds formed between the polymer chains [21]. Typical examples are chitosan-poly (ethylene glycol) hydrogel [22], chitosan-hyaluronic hydrogel [23], chitosan-glycerophosphate hydrogel [24], chitosan-alginate composite [25], chitosan-collagen composite [26] chitosan-hydroxyapatite composite [27] and chitosan-tricalcium phosphate composite [28]. They can be molded into different shapes and forms (films, fibers, sponges, beads and solutions). These materials are mainly applied in bone tissue engineering scaffold [29], drug delivery system [30], wound healing materials [31] and metal and dye absorbent for polluted water [32].

**Table 1:** Physiochemical characteristics of chitosan and their methods of determination.

Physiochemical Characteristics	Method of Determination
molecular weight	viscometry; gel permeation chromatography; light scattering; high performance liquid chromatography; matrix-assisted laser desorption/ionization-mass spectrometer
degree of deacetylation	infrared spectroscopy; ultra violet spectrophotometry; nuclear magnetic resonance spectroscopy (^1^H-NMR and ^13^C-NMR); conductometric titration; potentiometric tiltration; differential scanning calorimetry
crystallinity	X-ray diffraction

## 3. Bioactivities of Chitosan

### 3.1. Antibacterial Activity

Many reports have shown that chitosan exhibited antimicrobial activity, but the actual mechanism has not yet been fully elucidated. Several hypotheses have been proposed based on its cationic nature. Low-molecular-weight chitosan can penetrate bacterial cell walls, bind with DNA and inhibit DNA transcription and mRNA synthesis [33], while high-molecular-weight chitosan can bind to the negatively charged components on the bacterial cell wall. It forms an impermeable layer around the cell, changes cell permeability and blocks transport into the cell [34]. This hypothesis was further supported by the studies from Muzzarelli *et al.* [35]. The hydrophilicity and negative charge on the cell surface were higher on gram-negative bacterial cell walls than those of gram-positive bacteria. Thus the gram-negative bacteria showed a stronger interaction with chitosan, which resulted in stronger antibacterial activity against them. It was also reported that the amount of chitosan binding to the bacterial cell wall was dependent on the environmental pH value, molecular weight and degree of acetylation of chitosan. Low environmental pH increases the positive charge in the chitosan polymer, which favors binding to the bacterial cell wall [36]. Younes *et al.* [37] reported that a lower degree of chitosan acetylation and a lower pH are favorable to the antibacterial activity of chitosan. A reduction in the molecular weight of chitosan increases the antibacterial activity of chitosan toward Gram-negative bacteria and reduces the activity on the Gram-positive bacteria. Chitosan has a wide spectrum of activity and high killing rate against Gram-positive and Gram-negative bacteria. The activity is the result of interactions between chitosan and its derivatives with bacterial cell wall molecules. The studies mentioned in this section demonstrated a close relationship between the antibacterial activity and the hydrophilicity of the cell wall, thus the action is specific and showed lower toxicity toward mammalian cells [38].

Eco-friendly, low-cost and biocompatible composites prepared by immobilizing ZnO nanoparticles on the chitosan matrix by an *in situ* sol-gel conversion of precursor molecules in a single step demonstrates more potent antimicrobial activity against the Gram positive bacterium *Staphylococcus aureus* and the Gram negative bacterium *Escherichia coli* than chitosan. [39]. Chitosan solution exhibited higher antibacterial potency than chitosan submicroparticles toward both planktonic and biofilm-related antibiotic-resistant *Pseudomonas aeruginosa* cells isolated from chronic diabetic foot infections [40]. Low-molecular-weight water-soluble β-chitosan (with molecular masses of 5 and 10 kDa, respectively) exhibited potent antibacterial activity, even at pH 7.4, targeting the bacterial membrane, bringing about calcein efflux in artificial mimetic bacterial membrane and morphological alterations on bacterial surfaces. Data from an *in vivo* experiment employing a mouse model of bacterial infection provided evidence that low-molecular-weight water-soluble β-chitosan may find anti-infective and wound healing applications [41]. Quaternary ammonium chitosan/polyvinyl alcohol/polyethylene oxide hydrogels prepared with the use of gamma radiation exhibited desirable swelling ability, water evaporation rate as well as mechanical characteristics and high antibacterial potency against *E. coli* and *S. aureus*. The hydrogels are promising for use as wound dressing [42]. Chitosan/lignosulfonates multilayers modified fibers with chitosan in the outermost layer exhibited higher antimicrobial activity against *E. coli* and higher antioxidant activity than that of original fibers under the same oxidation conditions [43]. A method for reductive aminating of chitosan biopolymers to produce *N*,*N*-dialkyl chitosan derivatives was developed by employing as a precursor 3, 6-O-di-tert-butyldimethylsilylchitosan. The corresponding mono *N*-alkyl derivatives were synthesized, and all alkyl compounds were then quaternized. These derivatives were studied for antibacterial activity against Gram positive *S. aureus* and *Enterococcus faecalis*, and Gram negative *E. coli* and *P. aeruginosa*, which could be correlated to the length of the alkyl chain, but the order was dependent on the bacterial strain. Toxicity against human red blood cells and human epithelial Caco-2 cells was proportional to the length of the alkyl chain. The most active chitosan derivatives were found to be more selective for killing bacteria than the quaternary ammonium disinfectants cetylpyridinium chloride and benzalkonium chloride, along with the antimicrobial peptides melittin and LL-37 [44]. Nontoxic honey-polyvinyl alcohol-chitosan nanofibers were constructed with high honey concentrations (up to 40%) in addition to high chitosan concentrations (up to 5.5% of the total fiber weight) employing biocompatible solvents (1% acetic acid), chemically cross-linked with the help of glutaraldehyde vapor, as well as physically cross-linked by heating and freezing/thawing. The nanofibers demonstrated potent antibacterial activity against *S. aureus* but meager antibacterial activity against *E. coli* [45].

### 3.2. Antifungal Activity

The effects of molecular weight and degree of acetylation of chitosan on its antifungal activity varies with the fungus [37]. Chitosan was also found to exhibit antifungal activity against several phytopathogenic fungi such as *Penicillium* sp. in citrus fruit [46], *Botrytis cinerea* in cucumber plants [47], *Phytophthora infestans* [48], *Alternaria solani* and *Fusarium oxysporum* [49] in tomatoes. The suggested mechanism involved a permeable chitosan film formed on the crop surface which interfered with the fungal growth and activated several defense processes like chitinase accumulation, proteinase inhibitor synthesis, callus synthesis and lignification [50].

Silver nanoparticles distributed superficially on and internally in chitosan spheres demonstrated a macroporous feature, and could find applications such as fungicidal agents [51].

Chitosan applied at a dosage of 100 μg/mL killed the bulk of fungal species pathogenic to human pathogens examined but did not express toxicity to HEK293 and COS7 mammalian cells. Survival of *Galleria mellonella* larvae after infection with *C. albicans* was elevated by chitosan [52]. 

Water-soluble chitosan was fungistatic to *Macrophomina phaseolina*. Fungal infection was suppressed and the activities of enzymes associated with defense including chitosanase and peroxidase in infected seedlings were upregulated following exposure to water-soluble chitosan [53].

The existence of cranberry and quince juice in the composition of chitosan and whey protein-chitosan films reinforced the elasticity and reduced the tensile strength of the films. Chitosan and whey proteins-chitosan films with quince and cranberry juice added are potentially useful for increasing the shelf life of apples [54].

The fungal inhibition indices of deacetylated chitosans generally increased with a rise in molecular weight. Nevertheless, high-molecular-weight chitosan derivatives with a low hydrophobicity and low-molecular-weight derivatives with a high hydrophobicity displayed the highest potency in suppressing growth in *Aspergillus flavus in vitro* [55].

Cu-chitosan nanoparticles impeded spore germination and mycelial proliferation in *Fusarium oxysporum* and *Alternaria solani in vitro* and controlled Fusarium wilt and early blight in pot experiments [49]. 

### 3.3. Anti-HIV-1 Activity and for Construction of Nanoparticles Loaded with Anti-HIV Drugs

QMW-chitosan oligomers and WMQ-chitosan oligomers (in which Q, M and W stand for glutamine, methionine and tryptophan, respectively) exerted a protective action on C8166 cells against cytolytic effects of HIV-1RF strain. The oligomers suppressed syncytium formation induced by HIV, and reduced the HIV load without any inhibitory effects on activities of HIV-1 reverse transcriptase and protease *in vitro*. Syncytium formation was suppressed when HIV-infected and uninfected C8166 cells were co-cultured. QMW-chitosan oligomers and WMQ-chitosan oligomers inhibit HIV-induced cytopathic effects by exerting their effects on HIV entry stage [56]. Chitooligosaccharides are nontoxic and water-soluble compounds derived from chitosans by enzymatic degradation. Sulfated chitooligosaccharide III with a molecular weight of 3–5 kDa potently suppressed HIV-1 replication, HIV-1-induced syncytium formation, lytic action, and p24 antigen production. Sulfated chitooligosaccharide III obstructed viral entry and virus-cell fusion probably by interfering with the binding of HIV-1 gp120 to CD4 cell surface receptor. Unsulfated chitooligosaccharides did not have similar actions [57].

Chitosan-thioglycolic acid-conjugated nanoparticles loaded with the anti-HIV drug tenofovir show better biophysical characteristics for mucoadhesion than native chitosan nanoparticles [58]. Lamivudine-PEGylated chitosan drug, with a low cytotoxicity and a high potency, suppresses replication of HIV more powerfully than lamivudine [59]. Stable intravaginal mucoadhesive microspheres of tenofovir disoproxil fumarate have been formulated using chitosan as the matrix-forming mucoadhesive polymer [60]. Chitosan nanoparticles loaded with the anti-HIV drug saquinavir had excellent drug loading potential with high cell targeting efficiency resulting in efficient control of HIV proliferation in target T-cells. The findings reveal the potential of chitosan nanocarriers as vehicles for anti-HIV-1 drugs [61]. Tenofovir, an acyclic nucleoside phosphonate analog employed for anti-HIV therapy embedded into a type of nanocarriers based on poly-(d,l-lactide-co-glycolide) and/or chitosan, is a good anti-HIV drug carrier for investigating cellular uptake and drug delivery in target cells like macrophages [62]. Chitosan based nanoparticles loaded with tenofovir did not exhibit cytotoxicity to vaginal epithelial cells and *Lactobacillus crispatus*. Mucoadhesion increased as the nanoparticle diameter was reduced. However, the combined action of drug encapsulation efficiency and percent mucoadhesion for larger size nanoparticles yielded the best results. Hence, large-sized chitosan nanoparticles loaded with an anti-HIV drug appeared to be promising for stopping HIV transmission [63]. Chitosan-*O*-isopropyl-5′-*O*-d4T monophosphate conjugate nano-prodrugs may be deployed as targeting and sustained polymeric prodrugs for improving anti-HIV treatment and curtailing side effects of the treatment [64]. Glutaraldehyde cross-linked chitosan microspheres have been utilized for controlled delivery of zidovudine [65].

Chitosan and its derivatives have been shown to have antimicrobial activities and the results are summarized in Table 2.

**Table 2:** The antimicrobial activities of chitosan and its derivatives

Targets		Chitosan or Its Derivatives/MIC in μg/mL Reference
Gram-negative bacteria	*Escherichia coli*	chitosan 0.025% [38]; chitosan-Zn complex 0.00313% [38]; á-chitosan 9 μg/mL [41]; â-chitosan 9 μg/mL [41]; *N*,*N*-diethyl-*N*-methylchitosan 16 μg/mL [44]; *N*,*N*-dihexyl-*N*-methylchitosan 16 μg/mL [44]
	*E. coli* K88	chitosan 8 μg/mL [38]; chitosan nanoparticles 0.0625 μg/mL [38]; Cu loaded chitosan nanoparticles 0.0313 μg/mL [38]
	*E. coli* ATCC 25922	chitosan 8 μg/mL [38]; chitosan nanoparticles 0.0313 μg/mL [38]; Cu loaded chitosan nanoparticles 0.0313 μg/mL [38]
	*E. coli* O157	á-chitosan 9 μg/mL [41]; â-chitosan 9 μg/mL [41]
	*Pseudomonas aeruginosa*	chitosan 0.0125% [38]; chitosan-Zn complex 0.00625% [38]; á-chitosan 9 μg/mL [41]; â-chitosan 9 μg/mL [41]; *N*,*N*-diethyl-*N*-methylchitosan 32 μg/mL [44]
	*Proteus mirabilis*	chitosan 0.025% [38]; chitosan-Zn complex 0.00625% [38]
	*Salmonella enteritidis*	chitosan 0.05% [38]; chitosan-Zn complex 0.00625% [38]
	*S. choleraesuis* ATCC 50020	chitosan 16 μg/mL [38]; chitosan nanoparticles 0.0625 μg/mL [38]; Cu loaded chitosan nanoparticles 0.0313 μg/mL [38]
	*S. typhimurium*	á-chitosan 5 μg/mL [41]; â-chitosan 9 μg/mL [41]
	*S. typhimurium* ATCC 50013	chitosan 16 μg/mL [38]; chitosan nanoparticles 0.125 μg/mL [38]; Cu loaded chitosan nanoparticles 0.0625 μg/mL [38]
Gram-negative bacteria	*Enterobacter aerogenes*	chitosan 0.05% [38]; chitosan-Zn complex 0.00625% [38]
	*Listeria monocytogenes*	á-chitosan 9 μg/mL [41]; â-chitosan 9 μg/mL [41]
Gram-positive bacteria	*Staphylococcus aureus*	chitosan 0.05% [38]; chitosan-Zn complex 0.00625% [38]; á-chitosan 9 μg/mL [41]; â-chitosan 9 μg/mL [41]; *N*-ethyl-*N*,*N*-dimethylchitosan 4 μg/mL [44]
	*S. aureus* ATCC 25923	chitosan 8 μg/mL [38]; chitosan nanoparticles 0.125 μg/mL [38]; Cu loaded chitosan nanoparticles 0.0625 μg/mL [38]
	*Corynebacterium*	chitosan 0.025% [38]; chitosan-Zn complex 0.0313% [38]
	*Staphylococcus epidermidis*	chitosan 0.025% [38]; chitosan-Zn complex 0.0125% [38]; á-chitosan 5 μg/mL [41]; â-chitosan 5 μg/mL [41]
	*Enterococcus faecalis*	chitosan 0.05% [38]; chitosan-Zn complex 0.0125% [38]; *N*,*N*-diethyl-*N*-methylchitosan 16 μg/mL [44]
	*Bacillus cereus*	á-chitosan 9 μg/mL [41]; â-chitosan 9 μg/mL [41]
	*Bacillus megaterium*	á-chitosan 9 μg/mL [41]; â-chitosan 9 μg/mL [41]
Fungi	*Candida albicans*	chitosan 0.1% [38]; chitosan-Zn complex 0.1% [38]; chitosan 5 μg/mL [52]
	*Candida parapsilosis*	chitosan 0.1% [38]; chitosan-Zn complex 0.05% [38]; chitosan 40 μg/mL [52]
	*Candida krusei*	chitosan 5 μg/mL [52]
	*Candida glabrata*	chitosan 20 μg/mL [52]
	*Penicillium digitatum*	chitosan 65 μg/mL [46]
	*Penicillium italicum*	chitosan 57.5 μg/mL [46]
	*Fusarium proliferatum*	chitosan 2.5 μg/mL [52]
	*Hamigera avellanea*	chitosan 2.5 μg/mL [52]
	*Aspergillus fumigatus*	chitosan 1 μg/mL [52]
	*Rhizopus stolonifer*	chitosan 100 μg/mL [52]
	*Cryptococcus neoformans*	chitosan 5 μg/mL [52]
	*Cryptococcus gatti*	chitosan 2.5 μg/mL [52]
	*Macrophomina phaseolina*	chitosan 12.5 mg/mL [53]
Virus	IC_50_ of cytopathic effect by HIV-1_RF_	QMW-chitosan oligomers 48.14 μg/mL [56]
	IC_50_ of cytopathic effect by HIV-1_IIIB_	WMQ-chitosan oligomers 48.01 μg/mL [56]
	IC_50_ of p24 production by HIV-1_IIIB_	QMW-chitosan oligomers 67.35 μg/mL [56]
	IC_50_ of p24 production by HIV-1_Ba-L_	WMQ-chitosan oligomers 98.73 μg/mL [56]
	IC_50_ of p24 production by HIV-1_RTMDR1_	QMW-chitosan oligomers 81.03 μg/mL [56]; WMQ-chitosan oligomers 144.02 μg/mL [56]
	IC_50_ of luciferase expression by HIV-1_RF_	QMW-chitosan oligomers 68.13 μg/mL [56]; WMQ-chitosan oligomers 163.94 μg/mL [56]
	IC_50_ of interaction between gp41 and CD4 by HIV-1	QMW-chitosan oligomers 39.13 μg/mL [56]; WMQ-chitosan oligomers 51.48 μg/mL [56]
	IC_50_ of syncytia formation by HIV-1_RF_	sulfated chitooligosaccharides 2.19 μg/mL [57]
	EC_50_ of protection of lytic effect by HIV-1	sulfated chitooligosaccharides 1.43 μg/mL [57]
	IC_50_ of p24 production by HIV-1_RF_	sulfated chitooligosaccharides 4.33 μg/mL [57]
	IC_50_ of p24 production by HIV-1_Ba-L_	sulfated chitooligosaccharides 7.76 μg/mL [57]

### 3.4. Antitumor Activity

Recent investigations revealed that chitosan and its derivatives exhibited antitumor activity in both *in vitro* and *in vivo* models. Tokoro *et al.* [66] observed that the antitumor effect of chitosan derivatives was due to the increase in secretion of interleukin-1 and 2 which caused maturation and infiltration of cytolytic T-lymphocytes. It was further supported by Dass and Choong [67] whose *in vivo* study demonstrated that chitosan elevated lymphokine production and proliferation of cytolytic T-lymphocytes. Other investigations showed that chitosan was involved in direct killing of tumor cells by inducing apoptosis. Chitosan was shown to inhibit adhesion of primary melanoma A375 cells and proliferation of primary melanoma SKMEL28 cells. It also exhibited strong pro-apoptotic effects against metastatic melanoma RPMI7951 cells through inhibition of specific caspases, up-regulation of Bax and down-regulation of Bcl-XL proteins. Besides, it induced CD95 receptor expression on RPMI7951 surface, making them more vulnerable to FasL-induced apoptosis [68]. He *et al.* [69] reported that carboxymethylated chitosan protected the peripheral nerves and inhibited the apoptosis of cultured Schwann cells. They used the hydrogen peroxide- induced apoptosis model. Decreases in caspase-3, -9 and Bax activities and increases in Bcl-2 activity were detected.

Carboxymethyl chitosan suppressed migration of human hepatoma cells BEL-7402 *in vitro* and murine hepatoma 22 cells *in vivo*. The expression of matrix metalloproteinase-9 (MMP-9) in BEL-7402 cells was downregulated and pulmonary metastases of hepatoma-22 in Kunming mice were curtailed. Reduction of the lung damage brought about by the metastasis of H22 cells was noted. The suppressive action of carboxymethyl chitosan could be ascribed partly to the attenuated vascular endothelial growth factor and E-selectin levels [70]. It has been reported how the degree of deacetylation and molecular mass of chitosan oligosaccharides, procured from enzymatic hydrolysis of high-molecular-weight chitosan, affected antitumor activity. Determination of the degree of deacetylation and molecular weights of chitosan oligosaccharides were conducted by means of matrix-assisted laser desorption/ionization-mass spectrometry. The chitosan oligosaccharides formed a mixture composed essentially of dimers (18.8%), trimers (24.8%), tetramers (24.9%), pentamers (17.7%), hexamers (7.1%), heptamers (3.3%), and octamers (3.4%). The chitosan oligosaccharides were resolved by gel filtration into two main fractions: (1) chitosan oligosaccharides, composed of glucosamine (*n*), *n* = 3–5 with 100% deacetylation; and (2) HOS, consisting of glucosamine (5) as the minimum residues and a varying number of *N*-acetylglucosamine (*n*), *n* = 1–2 with 87.5% deacetylation in random order. The concentrations required for chitosan oligosaccharides, chitooligosaccharides, and HOS to achieve 50% cell death in PC3 prostate cancer cells, A549 lung cancer cell, and HepG2 hepatoma cells, were 25 μg/mL, 25 μg/mL, and 50 μg/mL, respectively. The high- molecular-weight chitosan had about half the efficacy of chitosan oligosaccharides and chitooligosaccharides. These findings show that the molecular weight and degree of deacetylation of chitosanoligosaccharides are critical determinants for inhibiting growth of cancer cells [71]. Chitooligosaccharides, products of chitosan hydrolysis, manifest a diversity of biological functions. Proliferation of hepatocellular carcinoma HepG2 cells was attenuated, the percentage of cells in S-phase was diminished and the rate of DNA synthesis was reduced after treatment with chitooligosaccharides. Among the cycle-related genes, PCNA, cyclin A and CDK-2 were down-regulated whereas p21 was up-regulated. Chitooligosaccharides undermined the activity of metastatic related protein (MMP-9) in Lewis lung carcinoma cells. Chitooligosaccharides suppressed tumor growth of HepG2 xenografts in severe combined immune deficient mice. Chitooligosaccharides repressed tumor growth and reduced the number of lung metastasis colonies and prolonged the lifespan of Lewis lung carcinoma-bearing mice [72]. Table 3 summarizes the literature mentioned in this review. 

**Table 3:** A summary of antitumor activities of chitosan and its derivatives.

Compound	Target Cell Lines or *in vivo* Model	Results	Ref.
chitosan	meth-A solid tumor transplanted into BALB/c mice	increased production of interleukins 1 and 2, sequentially, leading to the manifestation of antitumor effect through proliferation of cytolytic T-lymphocytes with the optimum inhibition ratio at the dose of 10 mg/kg	[66]
chitosan	aberrant crypt tumor lesions in the colon of mice	elevated lymphokine production and proliferation of cytolytic T-lymphocytes at the dose of 5 mg/kg	[67]
chitosan	A375, SKMEL28, and RPMI7951 cell lines	chitosan was coated in culture wells in which cultures with A375, SKMEL28, and RPMI7951 cells were carried out.	[68]
		decreased adhesion of A375 cells	
		decreased proliferation of SKMEL28 cells	
		inhibited specific caspases, upregulated Bax and downregulated Bcl-2 and Bcl-XL in RPMI7951 cells	
		induced CD95 receptor expression in RPMI7951 cell surface which renders them more susceptible to FasL-induced apoptosis	
carboxymethyl chitosan	hydrogen peroxide induced apoptosis models of Schwann cells	The cell viability was improved in a dose-dependent manner with maximum effect of 2.02 ± 0.16 fold at the dose of 200 μg/mL carboxymethyl chitosan	[69]
		decreased caspase-3, -9 and Bax activities and increased Bcl-2 activity	
carboxymethyl chitosan	BEL-7402 cell line	reduced the expression of MMP-9 in a dose-dependent manner	[70]
	hepatoma-22 cells in Kunming mice	inhibited the lung metastasis mouse model with the highest inhibition of 66.56% at the dose of 300 mg/kg	
chitosan	PC3 A549 and HepG2 cell line	suppressed cancer cell growth of PC3 A549 and HepG2 cells for 50% cell death at 25 μg/mL, 25 μg/mL and 50 μg/mL, respectively	[71]
chitosan	HepG2 and LCC cell line	inhibited MMP-9 expression, reduced cells in S-phase and decreased the rate of DNA synthesis, upregulated p21 and downregulated PCNA, cyclin A and CDK-2 with the highest inhibition at the dose of 1 mg/kg	[72]
	HepG2 and LCC xenografts in mouse model	inhibited tumor growth and decreased the number of metastatic colonies at the dose of 500 mg/kg	

### 3.5. Antioxidant Activity

Antioxidants are well-known for their beneficial effects on health. They protect the body against reactive oxygen species, which exert oxidative damage to membrane lipids, protein and DNA. Much effort has been invested to investigate the antioxidant activity of chitosan and its derivatives in recent years [6]. Park *et al.* [73] reported the *in vitro* oxygen radicals scavenging activity in chitosan and its derivatives. Low–molecular-weight chitosans are more active in scavenging free radicals, such as hydroxyl, superoxide, alkyl and 2,2-diphenyl-1-picrylhydrazyl radicals. It was proposed that the mechanism is due to the reaction of unstable free radicals with amino and hydroxyl groups on the pyranose ring, which form the stable radicals [12].

**Figure 3:**
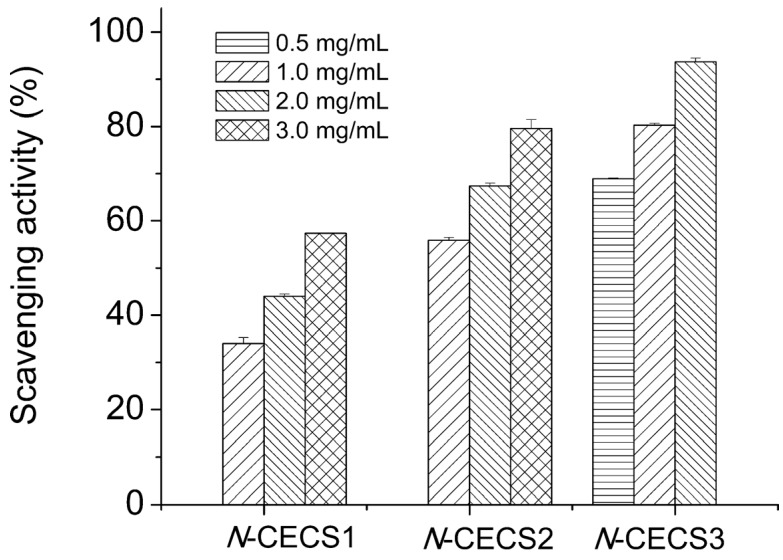
Scavenging effects of *N*-CECS with different degrees of substitution toward ABTS radicals. Reprinted with permission from [75] Copyright (2015) American Chemical Society.

Nine kinds of hetero-chitooligosaccharides with relatively higher molecular weights, medium molecular weights, and lower molecular weights have been prepared from partially deacetylated hetero-chitosans (90%, 75% and 50% deacetylated chitosan), and their scavenging activities against 1,1-diphenyl-2-picrylhydrazyl, carbon-centered hydroxyl, superoxide and radicals were studied by employing electron spin resonance spin-trapping technique. Carbon-centered, hydroxyl, and superoxide radicals were generated from 2,2-azobis-(2-amidinopropane)-hydrochloride, hydrogen peroxide-ferrous sulfate (Fenton reaction), and hypoxanthine-xanthine oxidase reaction. The electron spin resonance data demonstrated that medium-molecular-weight hetero-chitooligosaccharides prepared from 90% deacetylated chitosan manifested the highest radical scavenging potency. The radical-scavenging activity of hetero-chitooligosaccharides was related to the degree of deacetylation values and the molecular weight [74]. Chitosan was prepared by alkaline *N*-deacetylation of â-chitin from squid pens, and *N*-carboxyethylated derivatives (*N*-CESC) with different degrees of carboxyethyl group substitution (*N*-CESC3 possessed the highest degree of substitution while *N*-CESC1 possessed the lowest degree of substitution) were synthesized. All three *N*-CESC samples displayed good water solubility at pH above 6.5. They manifested potent 2,2′-azinobis(3-ethylbenzothiazoline-6-sulfonic acid) (ABTS) radical scavenging activity, with EC_50_ values under 2 mg/mL. The ABTS radical scavenging activities of *N*-CECS with different degrees of substitution and concentrations are shown in Figure 3. It showed that the activity of *N*-CECS toward ABTS increased with concentration. Besides, the addition of carboxyethyl groups to chitosan enhanced its radical scavenging activity against ABTS. The scavenging ability of *N*-CESC against superoxide radicals showed a good correlation with the degree of substitution and concentration of *N*-CESC. The data suggested that *N*-CESC can be utilized to produce chitosan derivatives with good biochemical characteristics *in vitro* [75]. Graft chitosan derivatives with low grafting percentages, produced by graft copolymerization of methacrylic acid sodium and acrylic acid sodium onto the etherification product of chitosan-carboxymethyl chitosan, exhibit a relatively low 50% inhibition concentration (IC_50_) for their radical scavenging activity, which could be attributed to their different contents of hydroxyl and amino groups in the polymer chains [76].

## 4. Applications

Chitosan and its derivatives are recognized as versatile biomaterials because of their diverse bioactivities, non-toxicity, biocompatibility, biodegradability and low-allergenicity. They have superior physical properties such as high surface area, porosity, tensile strength and conductivity. In addition, they can be easily molded into different shapes and forms (films, fibers, sponges, beads, powder, gel and solutions) [77].

### 4.1. Tissue Engineering

Tissue engineering describes the use of a combination of cells, engineering materials, and suitable biochemical factors to improve or replace biological functions. It includes a wide range of applications such as repair or replacement of part of or whole tissues (for example, bone, cartilage, blood vessels, bladder, skin and muscle). Chitosan-based biomaterials have become a popular target in development for tissue engineering and significant progress has been made recently. It provides certain mechanical and structural properties for proper functioning for the repaired tissues [78].

*N*-methacryloyl chitosan, produced as a result of a single-step chemoselective *N*-acylation reaction, acquires the desirable features of hydrosolubility, UV crosslinkability and injectability. It facilitates quick and cost-effective construction of patterned cell-loaded polysaccharide microgels with distinctive amino groups as building materials for tissue engineering and quick transdermal curing hydrogel *in vivo* for localized and sustained protein delivery [79]. Chitosan-â-tricalcium phosphate composite exhibited histocompatibility with Beagle mesenchymal stem cells and was devoid of an effect on cellular growth and proliferation. It manifested efficacy in enhancing osteogenesis and vascularization and repair of bone defects in conjunction with mesenchymal stem cells [80]. Reinforcement of silk matrix with chitosan microparticles (silk:chitosan 1:1, 1:2 and 2:1) produced a visco-elastic matrix that promoted redifferentiation of caprine chondrocytes and retained more glycosaminoglycan which enhanced the aggregate modulus of the construct similar to native tissue. The data revealed one step forward in optimizing the construction of biomaterial scaffolds for cartilage tissue engineering [81]. Bone morphogenic protein 2/Chitosan microspheres were successively loaded onto a deproteinized bovine bone scaffold. BMP-2 underwent an initial burst release followed by a sustained release. The encapsulated bone morphogenetic protein 2 possessed biological activity. Biocompatibility was good. The microsphere scaffold system may find applications in tissue engineering [82]. A chitosan hollow tube employed for regeneration of the injured rodent transected sciatic nerve yielded results comparable to autologous nerve graft repair [83]. Implantation of chitosan nanofiber tube could partially restore the function of a damaged phrenic nerve in beagle dogs as seen in improvement of diaphragm movement, slow phrenic nerve conduction, connection of the damaged nerve by newly regenerating nerve fibers surrounded by granulation tissue within the chitosan nanofiber tube [84]. The loss of spinal cord tissue and cavity formation impedes the repair of damage to the spinal cord. The scaffold of chitosan+ECM+SB216763 enhanced neural stem cell differentiation into neurons, oligodendrocytes and astrocytes and hence is promising for repair of damaged spinal cords [85]. Chitosan/bioactive glass nanoparticles scaffolds possess the shape memory characteristics of chitosan and the biomineralization activity of bioactive glass nanoparticles for applications in bone regeneration [86].

### 4.2. Drug Delivery System

Chitosan has been widely used in pharmaceutical industry in drug delivery systems in different forms, like tablets, microspheres, micelles, vaccines, nucleic acids, hydrogels, nanoparticles and conjugates. Chitosan and its derivatives can be used in drug delivery systems in both implantable as well as injectable forms through oral, nasal and ocular routes. Besides, they facilitate transmucosal absorption which is important in nasal and oral delivery of some polar drugs like peptides along with protein vaccines for their administration [87]. It is commonly used as an excipient in tablet formulation for oral medication. High-molecular-weight chitosan is more viscous and delays the release of the active ingredient, prolongs the duration of drug activity, improves therapeutic efficiency as well as reducing the side effects of oral tablets [88]. Chitosan microspheres have been extensively investigated for controlled release of drugs and vaccines through oral and nasal delivery. They were prepared by complexation between the cationic chitosan in addition to the anionic compounds such as tripolyphosphate or alginates [89]. Different drugs or vaccines were loaded in the microspheres, they were protected, especially drugs which are protein in nature, in the digestive tract [90] and absorbed through the paracellular route on the epithelial layer [91]. The surface activity of chitosan is low as it does not possess any hydrophobic portions. It can be improved by chemical modifications at its glucosidic group with a hydrophobic substituent. The chitosan form micelles with an external hydrophilic shield and an internal hydrophobic center [92]. The hydrophobic drugs were protected in the center with improved solubility and bioavailability. The chitosan micelles were formed by the electrostatic repulsions between oppositely charged polymers [93]. The chitosan hydrogels are three-dimensionally structured hydrophilic polymers which can absorb and hold up to thousands of times more fluids than their dry weights and use in drug delivery. The drugs are loaded in the hydrogels by diffusion, entrapment, and tethering. Usually, the loaded hydrogel is injected into the body and the drug can diffuse into the neighboring tissues. The recent development of *in situ* forming depots using chitosan-based hydrogel has attracted much attention as a new method for controlled drug release [94]. The original thermosensitive chitosan-based polymer is in solution form at room temperature. When it is injected into the body, it forms semi-solid hydrogels at the physiological temperature. It showed protection for the drugs from physiological degradation, combined with prolonged and steady release of drugs [95]. Chitosan nanoparticles exhibit outstanding biodegradable and biocompatible properties which have been studied extensively as drug carriers. They can be administered through non-invasive means such as ocular, nasal, oral, and pulmonary routes. The drugs are protected from chemical and enzymatic degradation in the digestive system. Besides, they bind strongly to mucus which enhances the adsorption through intestinal epithelial cells [96]. They can be prepared by a number of methods such as ionotropic gelation, emulsion cross-linking, emulsion-solvent extraction, emulsification solvent diffusion, emulsion-droplet coalescence, complex coacervation, reverse microemulsion technique in addition to self-assembly [97]. The most common method is ionotropic gelation method in which the preparation conditions are mild and less time-consuming. It is based on spontaneous aggregation of positively-charged chitosan with a negatively-charged sodium polymer such as tripolyphosphate. The drugs are dissolved in either component. Then a nanoparticle suspension is formed upon addition of the other component under vigorous agitation [98]. The chitosan derivatives conjugate with antitumor agents to form a good partner for targeted drug delivery in cancer treatment. They manifest reduced side effects compared with the original drug because of a predominant distribution into the cancer cells and a progressive release of the free drug from the conjugates [99].

An intermolecular complex formed from a 1:1 ratio by weight of 30-kDa chitosan and sulfobutyl ether â-cyclodextrin was less soluble than either component. At pH 1.2, the drug famotidine was slowly released from the less-soluble chitosan/sulfobutyl ether â-cyclodextrin complex formed superficially on the tablet upon exposure to water, followed by dissolution of the interpolymer complex and, finally, breakdown of the tablet. At pH 6.8, gel formation by chitosan accounted for the gradual release. The slow release of the tablet was seen in the drug absorption *in vivo* following treatment of rats via the oral route [100]. Glycol chitosan nanogel uptake took place essentially by means of endocytosis mediated by flotillin-1 and Cdc42 and macropinocytosis with the participation of the actin cytoskeleton, and internalization mechanisms through the folate receptor. About half of the nanogel population was found in endolysosomal compartments, while the rest was located in undefined cytoplasmic compartments at the end of seven hours of incubation with HeLa cells. Glycol chitosan nanogels may be useful as drug delivery vectors for targeting different intracellular compartments [101]. Controlled-release, floating and mucoadhesive beads of glipizide were developed by polyionic complexation technique using chitosan as cationic and xanthan gum as anionic polymers. The beads displayed pH-dependent swelling kinetics, good bioadhesive strength and comparable floating capacity in the gastric fluids. Altering the chitosan to xanthan gum ratio did not affect the drug release [102]. Insulin was physically and chemically stable in a polyelectrolyte complex composed of insulin and 13-kDa low-molecular-weight chitosan in Tris-buffer (pH 6.5). Solubilization of the insulin-low-molecular-weight chitosan polyelectrolyte complex in a reverse micelle system, given to hyperglycemic rats, constituted an oral bioactive insulin delivery system [103]. The colon is a drug delivery target because of the long transit time and thus a prolonged drug absorption time. Progesterone has an abbreviated half-life, much first-pass metabolism, and low oral bioavailability. An oral Zn-pectinate/chitosan multiparticulate system prepared by ionotropic gelation, allowing increased oral bioavailability of progesterone as well as progesterone residence time in plasma for colonic-specific progesterone delivery, was developed. Negligible progesterone release in simulated gastric fluids was observed, but there was a burst release at pH 6.8 and sustained release at pH 7.4 [104]. Incorporation of glutaraldehyde augmented the drug entrapment efficacy of the Boswellia resin-chitosan polymer composites in phosphate buffer (pH 6.8). The drug release rate surged to 92% as the gum resin concentration in the composites was elevated to 80%. The composites released 60%–68% drug load in seven hours [105]. Water in oil nanosized systems containing low-molecular-weight chitosan-insulin polyelectrolyte complexes were constructed and their hypoglycemic activity was assayed in diabetic rats. The 1.3-kDa chitosan with 55% (among 55%, 80% and 100%) deacetylation possessed the most potent hypoglycemic activity among three molecular weights (namely, 1.3, 13 and 18 kDa) and different extents of deacetylation [106]. Higher retention of conjugate formed between chitosan and catechol derived from mussel adhesive proteins, in the gastrointestinal tract *versus* unmodified chitosan, owing to production of irreversible catechol mediated-crosslinking with mucin, may be advantageous for production of new mucoadhesive polymers to be employed for mucosal drug delivery [107]. Microcapsules loaded with fish oil were produced from oil-in-water emulsions by both membrane emulsification and ultrasonic emulsification employing *N*-stearoyl *O*-butylglyceryl chitosan as shell material. The microcapsules produced by membrane emulsification displayed a larger diameter and more desirable loading capacity and encapsulation efficiency. Microcapsules from both membrane emulsification and ultrasonic emulsification demonstrated sustained release of fish oil which had higher thermostability [108]. 

### 4.3. Wound Healing

Chitosan and its derivatives exhibit biodegradable, biocompatible, antimicrobial activity and low immunogenicity which are advantageous for development as biomaterials for wound healing [36]. They provide a three-dimensional tissue growth matrix, activate macrophage activity and stimulate cell proliferation [109]. Chitosan promotes activity of polymorphonuclear leukocytes, macrophages and fibroblasts that enhance granulation as well as the organization of the repaired tissues [110]. It will be slowly degraded into *N*-acetyl-β-d-glucosamine which stimulates fibroblast proliferation, aids regular collagen deposition in addition to stimulating hyaluronic acid synthesis at the wound site. It accelerates the healing progress along with preventing scar formation [111].

Nanofibrous and adhesive-based chitosan have been developed as wound dressing materials recently [112]. The electrospun chitosan nanofiber mats were found to be porous, have a high tensile strength, high surface area of the mats combined with ideal water vapor and oxygen transmission rate. It also showed compatibility with adipose derived stem cells, which is considered beneficial for wound healing [113]. The adhesive-based wound dressing was usually applied in surgery to enhance wound healing. The chitosan adhesive shows strong sealing strength as well as not requiring sutures or staples. It can effectively stop bleeding from blood vessels along with air leakage from the lung [114]. 

**Figure 4:**
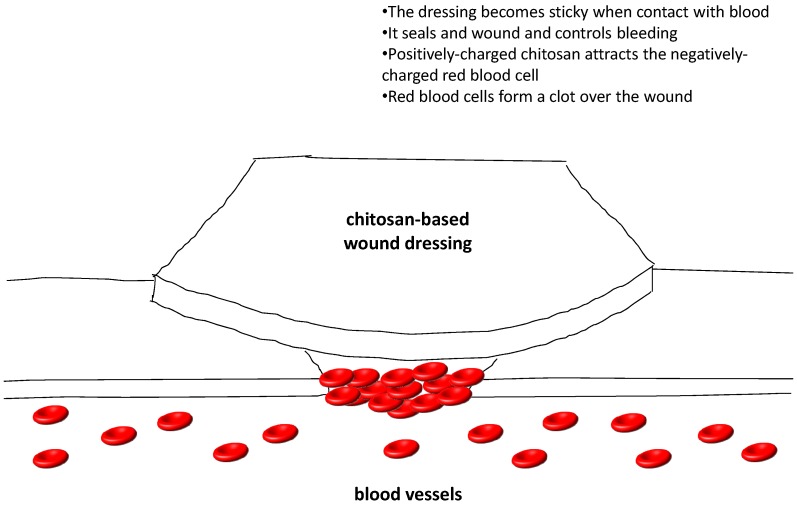
A diagrammatic presentation of how the chitosan-based wound dressing works.

Several clinical studies have reported the favorable results of using chitosan for wound healing in patients undergoing plastic surgery [115], skin grafting [116,117] and endoscopic sinus surgery [118]. Currently, there are a number of chitosan-based wound dressings available in the market in the form of non-wovens, nanofibers, composites, films, and sponges. HemCon^®^ hemostatic bandages which are chitosan-coated are the most famous. They were widely used in treating external hemorrhage in military operations as well as pre-hospital bleeding cases [119]. Similar products like GuardaCare^®^, ChitoFlex^®^ and ChitoGauze^®^ are temporary surgical dressings, stuffable dressings and gauze dressings, respectively, and are products from the same company. All of them offer an antibacterial barrier. Another chitosan-coated hemostatic gauze, Celox™ Gauze, has also been found to be effective in emergency bleeding control. They showed efficacious hemostasis in penetrating limb trauma when compared with the conventional pressure bandage in clinical trials [120]. Celox™ Granules, in the form of flakes, work in the same way as the gauze. When Celox™ gets into contact with blood, it swells and sticks together to form a gel-like clot. It works independently from the blood clotting mechanism and works well with hypothemic as well as heparinized blood. Celox™ and ChitoGauze^®^ are approved for use by US military as hemostatic agents [119]. Chito-Seal™ and Clo-Sur^PLUS^ PAD are topical hemostasis chitosan-coated pads used for promoting vascular hemostasis following percutaneous catheters or tubes interventional. When put on the puncture site, the positively charged chitosan attracts the negatively charged red blood cells as well as platelets, thus shortening the clot formation in addition to hemostasis time [121]. Tegasorb™ and Tegaderm™ wound dressings are used for protective treatment of partial and full thickness dermal ulcers, leg ulcers, superficial wounds, abrasions, burns, as well as donor sites. The chitosan presented absorbs wound exudates, then swells up and produces a soft gel mass that enhances wound healing [122]. Other chitosan-containing wound-care products available on the market include ChiGel, Chitopack C^®^ and TraumaStat™. Figure 4 shows a diagrammatic presentation of how the chitosan-based wound dressing works. 

Hydrogel films based on carboxyl-modified poly(vinyl alcohol) and chitosan were cross-linked through amide linkage formation. Their mechanical properties in dry and swollen state were greatly improved with high swelling ratio. They could maintain a moist environment over wound bed. Cross-linked hydrogel films loaded with gentamicin sulfate displayed sustained drug release, in addition to effectively suppressing bacterial proliferation along with protecting the wound from infection [123]. Curcumin-encapsulated bioglass-chitosan, which is promising for wound healing applications, displayed higher 1,1-diphenyl-2-picrylhydrazyl and superoxide free radical quenching activities compared with unmodified curcumin, antibacterial activity against *Staphylococcus aureus*, and reduction in tumor necrosis factor- â production [124]. 

### 4.4. Water Treatment

Chitosan is regarded as one of the most efficient materials for adsorption of pollutants in water treatment systems. The presence of amino and hydroxyl groups in chitosan allows its adsorption interactions with pollutants such as dyes [125], metals [126] and organic compounds [127], *etc.* Besides, these functional groups are subjected to modifications (cross-linking and grafting) which enhance the absorption efficiency and specificity [128]. For example, cross-linking the functional groups of chitosan improves the adsorption efficiency of chitosan at low pH. Grafting with sulphur or nitrogen improves specificity and capacity for some metal ions [129]. The dye adsorption performance by unmodified chitosan is good; however, its low stability has prompted many researchers to consider modifying them. Different modifications (grafted amino group, carboxyl group, sulfur group and alkyl group; cross-linked epichlorohydrin, ethylene glycol diglycidyl ether, glutaraldehyde and tripolyphosphate) were studied and employed to improve the adsorption efficiency as well as the mechanical and physical properties. The original properties of chitosan have been altered and are more suitable for the adsorption of different dyes [125]. Chitosan can act as a chelating polymer for binding toxic heavy metal ions. Metal cations can be chelated by the amine groups of chitosan in near-neutral conditions. For metal anions, the adsorption depends on electrostatic attraction on protonated amine groups in acidic conditions. Chitosan modified with different derivatives offers a wide range of properties for specific adsorption of metal ions [126]. Organic pollutants, including phenolic compounds, polycyclic aromatic hydrocarbons, organic pesticides and herbicides, cause health and environmental problems due to their toxic effects coupled with poor biodegradability. Chitosan adsorption for organic pollutants offer high adsorption capacities, insensitivity to toxic substances, good modifiability as well as recoverability. The mechanism depends on the characteristics along with the nature of the pollutants and is complicated. There is no simple theory or single mechanism to explain adsorption characteristics. Some interactions related to the adsorption mechanism include partition, diffusion, cation exchange, hydrogen bond, Van der Waals force, dipole–dipole interactions and electrostatic interaction [127].

Quaternary tetraalkylammonium chitosan derivatives can be utilized in the form of an inexpensive perchlorate-specific solid-phase extraction anion exchange cartridge in conjunction with colorimetric analysis for perchlorate removal or analysis [130]. Elevated initial fluoride concentrations, low dosages, combined with low ambient temperatures promoted, by ion exchange between chloride and fluoride, relatively selective fluoride adsorption from aqueous solutions on an Fe-impregnated chitosan granular adsorbent containing Fe-chelated to amino and hydroxyl groups on chitosan. At low fluoride concentrations, the adsorption characteristics followed the Langmuir model and at high initial fluoride concentrations, it followed the Freundlich model [131]. Magnetic hydroxypropyl chitosan/oxidized multiwalled carbon nanotubes composites were good for elimination of lead ions from aqueous solutions with pseudo-second-order kinetics. The optimal contact time and pH were 120 min and pH 5.0, respectively. Sips model is more appropriate than Langmuir, Freundlich and Dubinin-Radushkevich models for describing the adsorption process, which was endothermic and spontaneous [132]. Protonated polyamidoamine grafted chitosan beads loaded with Zr(IV) ions, produced by amination of chitosan beads by ethylenediamine through Michael addition and followed by protonation, eliminated fluoride ions from aqueous solutions with higher selectivity than other metal ions. The adsorption was spontaneous and endothermic [133]. Chitosan nanofibers formed by electrospinning with 5% chitosan in acetic acid as spinning solution were cross-linked with glutaraldehyde to eliminate chromium from water by adsorption with a pseudo-second order kinetic model, following a mixed isotherm of Freundlich and Langmuir. The maximum nanofiber adsorption capacity was more than double that of chitosan powders. Sodium, calcium magnesium, nitrate, and chloride, but not sulfate ions, had nil or negligible effect on adsorption which involved hydroxyl as well as amino groups of chitosan [134]. Chitosan along with polyphenol oxidase were used in conjunction for eliminaton of bisphenol derivatives from aqueous solutions based on adsorption of enzymatically generated quinine derivatives on chitosan beads or chitosan powders. The optimum temperature and pH conditions were 40 °C and pH 7 for bisphenols B, E, O, and Z; 30 °C and pH 7 for bisphenols C and F; and 40 °C and pH 8 for bisphenol T. The removal time could be reduced by using more chitosan beads or chitosan powders of a smaller size [135]. Sorption of Cd(II) ions onto cross-linked low-molecular-weight chitosan pyruvic acid derivative followed Langmuir isotherm model and displayed pseudo second-order kinetics. Two levels of Cd(II) concentration (1 or 3 mg/L, temperature (45 or 70 °C) and solution pH (6.0 or 10.0) were considered. The factors and their interaction effect on the efficacy of cadmium elimination followed the order: Cd(II) concentration > solution pH > interaction between solution pH and Cd(II) concentration > interaction between solution pH, temperature and Cd(II) concentration [136]. A procedure for concurrent elimination of cyanobacterial harmful algal blooms and microcystins, which may pose a public health threat, was devised by employing chitosan-modified local soil flocculation and microbe-modified soil capping. Breakdown of toxin was a consequence of the joint actions of flocculation and microcystin-degrading bacteria in the capping material, which inhibits dilution of bacterial biomass, enriches the algal cells, sequesters the liberated toxins, and promotes toxin biodegradation [137]. Chitosan nanorod with a minimum particle size smaller than 100 nm was produced by crosslinking chitosan of low molecular weight with polyanion sodium tripolyphosphate and was then physicochemically characterized (using AFM, DSC, FT-IR, SEM, TGA and XRD) for waste water treatment. Its sorption capacity and sorption isotherms for chromium were studied. How the initial concentration of chromium ions, sorbent amount, duration of shaking and solution pH affected sorption capacity were also examined. The findings disclosed that in the solid state nanochitosan was rod shaped and it effectively sorbed chromium (VI) to chromium (III) ions. The sorption capacity of chitosan nanoparticles is remarkably high; additionally the adsorbent favors multilayer adsorption based on the Langmuir, the Freundlich and the Temkin sorption isotherms. The adsorption follows pseudo-second-order kinetics, which infers transformation of chromium (VI) to chromium (III). Hence nanochitosan is a good biosorbent for chromium removal from water [138].

### 4.5. Obesity Treatment

Chitosan is marketed as a dietary supplement or nutraceutical for lowering serum cholesterol and controlling obesity. It is not specifically digested in our gastrointestinal tract. Chitosan swells up giving the feeling of satiety by physically filling the stomach [139]. By inhibiting pancreatic lipase activity, chitosan can reduce the absorption of dietary fat in intestines. Besides, it can bind and precipitate fat in the intestines so that it is not absorbed. The cationic chitosan binds with anionic carboxyl groups of fatty acids and bile acids, it also interferes with emulsification of neutral lipids like cholesterol and other sterols by binding them with hydrophobic interaction, thus fat and cholesterol absorption from gastrointestinal tract is reduced [140]. However, when it was evaluated in clinical trials, the results varied. There is some support that chitosan is more effective than placebo in the short-term treatment of obesity and hypercholesterolemia [141,142,143,144]. However, results obtained from larger and better-controlled trials showed that the effect of chitosan on body weight and serum cholesterol appeared to be ineffective, unconvincing and devoid of any clinical significance [145,146,147].

### 4.6. Other Applications

#### 4.6.1. Cardiovascular Diseases Treatment

Administration of chitosan-oligosaccharides by gastric gavage to apolipoprotein E deficient mice (apoE-/-) fed a high fat diet for 12 weeks lowered triglyceride and cholesterol levels in non-high density lipoprotein fractions, undermined atherosclerosis, increased atherosclerotic plaque stability, upregulated hepatic expression of low density lipoprotein receptor, scavenger receptor BI and also the expression of macrophage scavenger receptor BI and ATP binding cassette transporter A1. There was no effect on the plasma lipid level in LDL-R mice with a deficiency of low density lipoprotein receptors and cholesterol absorption in wild-type mice [148].

#### 4.6.2. Treatment of Age-Related Diseases 

The potential utility of chitosan, chitooligosaccharides, their derivatives and chitosan-based functional food in forestalling and therapy of aging-associated diseases has been discussed and in this paper. The pathophysiological roles played by oxidative stress, oxidation of low density lipoprotein, increase of tissue stiffness, conformational changes of protein, aging-associated and chronic inflammation have been reviewed [149].

#### 4.6.3. Mucosal Immunity Enhancer

Nasal administration of *Bacillus anthracis* protective antigen adsorbed on chitosan -C48/80 nanoparticles into mice produced elevated serum titers of antibodies against protective antigen and a more balanced Th1/Th2 pattern compared with C48/80 in solution or chitosan/alginate -C48/80 nanoparticles. The incorporation of C48/80 within chitosan nanoparticles promoted a stronger mucosal immunity than other adjuvanted groups examined. The findings indicate that chitosan nanoparticles could act in concert with a mast cell activator to effect nasal immunization [150]. 

#### 4.6.4. Dry Mouth Syndrome Treatment

Chitosan-thioglycolic-mercaptonicotinamide conjugates, which were non-toxic against Caco-2 cells and synthesized by the oxidative S-S coupling of chitosan-thioglycolic acid with 6-mercaptonicotinamide, manifested improved swelling and cohesive characteristics compared with unmodified chitosan and were promising for therapy of dry mouth syndrome in which lubrication and mucoadhesiveness of the mucosa are wanting [151].

#### 4.6.5. Food Industry

The use of electrospun chitosan fibers for wrapping during dry-aging of beef for up to three weeks yielded better results with regard to decrease of counts of microorganisms, molds and, yeasts, yield, lighter appearance, and less muscle denaturation in comparison with traditional dry-ageing. Regarding the wet-aging of beef, there were little weight and trimming losses but growth of lactic acid bacteria was detected [152].

#### 4.6.6. Gene Silencing in Disease Vector Mosquito Larvae

Chitosan/interfering RNA nanoparticles mixed with food and consumed by larvae of *Aedes aegypti* (vector of the dengue and yellow fever) and *Anopheles gambiae* (vector of the primary African malaria vector) represented a methodology that can be applied to many insects and pests [153]. 

## 5. Conclusions

Chitosan is a biodegradable and inexpensive polymer which has numerous applications in biomedical as well as pharmaceutical industries. A large amount of research work has been done on chitosan and its derivatives for the purpose of tissue engineering, drug delivery, wound healing, water treatment, antitumor and antimicrobial effects. 

Chitosan and its derivatives have been widely studied for potential tissue engineering biomaterials as they will be degraded at a reasonable rate without causing any inflammatory reaction or producing any toxic end-products when the new tissues are formed. They are porous in nature for diffusion of gases, nutrients, and metabolic wastes for the seeded cells along with increasing the surface area for cell attachment, migration and differentiation. They can be molded easily into anatomical shape and volume; are biocompatible with the surrounding biological fluids and tissues; as well as providing temporary mechanical support. All these properties fit the special properties use as tissue engineering scaffold.

Chitosan demonstrated antitumor activity in terms of as a therapeutic agent and as a drug carrier. As a therapeutic agent, it has been suggested that the antitumor activity related to their ability to induce cytokines production through increased T-cell proliferation. Other investigators reported that it involved MMP-9 inhibition and strong pro-apoptotic effects against tumor cells. The details of their actions are summarized in Table 3. 

As a drug carrier, chitosan and its derivatives improve drug absorption, stabilize drug constituents for drug targeting in addition to drug release enhancement. Some studies showed that the antitumor agents/chitosan and its derivatives or conjugates exhibit better antitumor effects than the original form with a decrease in adverse effects. It is due to a predominant distribution in the cancer tissue and a gradual release of free drug from the conjugates.

Nowadays, the use of the wound-care products like hemostatic dressings is still uncommon due to the extra cost though they are more beneficial for fast recovery. However, using these products enables fewer changes of dressings and less attention to the patients, which can reduce the resources and workload required by the healthcare workers. 

The recently developed nano-chitosan adsorbent gave new hope of commercializing chitosan into an alternative adsorbent. Chitosan, as an adsorbent, exhibited specific characteristics such as inexpensiveness, environmentally friendliness, versatility, biodegradability, high adsorption capability and selectivity. The nano-sized materials display the advantages of high specific surface area, low internal diffusion resistance, small size and quantum size effect that could enable them to exhibit higher capacities for pollutants. Up to now, adsorption of a number of dyes and heavy metal ions using nano-chitosan has been studied. In order to develop a multipurpose adsorbent for industrial wastewater, organic pollutants and regeneration studies need to be done. These works can determine the reusability and versatility of nano-chitosan as an effective adsorbent. 

For obesity treatment, chitosan appears to be safe for consumption and there is no known side-effect. However, its claim for obesity treatment seems to be lack of scientific support and more studies need to be done on testing the validity of such claim.

Quaternized chitosan, which introduces permanent positively charged quaternary groups to the hydroxyl group or amino group of the polymers, enhances antimicrobial activity over a wide pH range. In addition, the quaternized chitosan can also be used as an antimicrobial coating in orthopedic and dental implants and an antimicrobial wound dressing material for surgery.

## References

[1] Chandy T., Sharma C.P. (1990). Chitosan-as a biomaterial. Biomater. Artif. Cells Artif. Organs.

[2] Venkatesan J., Kim S.K. (2010). Chitosan composites for bone tissue engineering—An overview. Mar. Drugs.

[3] Kumar M.N., Muzzarelli R.A., Muzzarelli C., Sashiwa H., Domb A.J. (2004). Chitosan chemistry and pharmaceutical perspectives. Chem. Rev..

[4] Karagozlu M.Z., Kim S.-K., Kim S.K. (2014). Chapter Twelve—Anticancer effects of chitin and chitosan derivatives. Advances in Food and Nutrition Research.

[5] Martins A.F., Facchi S.P., Follmann H.D., Pereira A.G., Rubira A.F., Muniz E.C. (2014). Antimicrobial activity of chitosan derivatives containing *N*-quaternized moieties in its backbone: a review. Int. J. Mol. Sci..

[6] Ngo D.H., Kim S.K., Kim S.K. (2014). Chapter Two—Antioxidant effects of chitin, chitosan, and their derivatives. Advances in Food and Nutrition Research.

[7] Aranaz I., Mengíbar M., Harris R., Paños I., Miralles B., Acosta N., Galed G., Heras Á. (2009). Functional characterization of chitin and chitosan. Curr. Chem. Biol..

[8] Onsosyen E., Skaugrud O. (1990). Metal recovery using chitosan. J. Chem. Technol. Biotechnol..

[9] Felt O., Buri P., Gurny R. (1998). Chitosan: A unique polysaccharide for drug delivery. Drug Dev. Ind. Pharm..

[10] Han L.K., Kimura Y., Okuda H. (1999). Reduction in fat storage during chitin-chitosan treatment in mice fed a high-fat diet. Int. J. Obes. Relat. Metab. Disord..

[11] Zhang Y., Zhang M. (2002). Three-dimensional macroporous calcium phosphate bioceramics with nested chitosan sponges for load-bearing bone implants. J. Biomed. Mater. Res..

[12] Younes I., Rinaudo M. (2015). Chitin and chitosan preparation from marine sources. Structure, properties and applications. Mar. Drugs.

[13] Percot A., Viton C., Domard A. (2003). Optimization of chitin extraction from shrimp shells. Biomacromolecules.

[14] Jung W.J., Jo G.H., Kuk J.H., Kim K.Y., Park R.D. (2006). Extraction of chitin from red crab shell waste by cofermentation with *Lactobacillus paracasei* subsp. tolerans KCTC-3074 and *Serratia marcescens* FS-3. Appl. Microbiol. Biotechnol..

[15] Kafetzopoulos D., Martinou A., Bouriotis V. (1993). Bioconversion of chitin to chitosan: Purification and characterization of chitin deacetylase from *Mucor rouxii*. Proc. Natl. Acad. Sci. USA.

[16] No H.K., Meyers S.P. (1995). Preparation and characterization of chitin and chitosan—A review. J. Aquat. Food Prod. Technol..

[17] Rege P.R., Block L.H. (1999). Chitosan processing: Influence of process parameters during acidic and alkaline hydrolysis and effect of the processing sequence on the resultant chitosan’s properties. Carbohyd. Res..

[18] Galed G., Miralles B., Inés Paños I., Santiago A., Heras Á. (2005). *N*-Deacetylation and depolymerization reactions of chitin/chitosan: Influence of the source of chitin. Carbohyd. Polym..

[19] Nwe N., Furuike T., Tamura H., Se-Kwon K. (2014). Chapter One—Isolation and characterization of chitin and chitosan from marine origin. Advances in Food and Nutrition Research.

[20] Philippova O.E., Korchagina E.V., Volkov E.V., Smirnov V.A., Khokhlov A.R., Rinaudo M. (2012). Aggregation of some water-soluble derivatives of chitin in aqueous solutions: Role of the degree of acetylation and effect of hydrogen bond breaker. Carbohyd. Polym..

[21] Bhattarai N., Gunn J., Zhang M. (2010). Chitosan-based hydrogels for controlled, localized drug delivery. Adv. Drug Deliv. Rev..

[22] Tsao C.T., Leung M., Chang J.Y., Zhang M. (2014). A simple material model to generate epidermal and dermal layers *in vitro* for skin regeneration. J. Mater. Chem. B Mater. Biol. Med..

[23] Lindborg B.A., Brekke J.H., Scott C.M., Chai Y.W., Ulrich C., Sandquist L., Kokkoli E., O’Brien T.D. (2015). A chitosan-hyaluronan-based hydrogel-hydrocolloid supports *in vitro* culture and differentiation of human mesenchymal stem/stromal cells. Tissue Eng. Part A.

[24] Salis A., Rassu G., Budai-Szucs M., Benzoni I., Csanyi E., Berko S., Maestri M., Dionigi P., Porcu E.P., Gavini E. (2015). Development of thermosensitive chitosan/glicerophospate injectable *in situ* gelling solutions for potential application in intraoperative fluorescence imaging and local therapy of hepatocellular carcinoma: A preliminary study. Expert Opin. Drug Deliv..

[25] Rahaiee S., Shojaosadati S.A., Hashemi M., Moini S., Razavi S.H. (2015). Improvement of crocin stability by biodegradeble nanoparticles of chitosan-alginate. Int. J. Biol. Macromol..

[26] Zeng W., Rong M., Hu X., Xiao W., Qi F., Huang J., Luo Z. (2014). Incorporation of chitosan microspheres into collagen-chitosan scaffolds for the controlled release of nerve growth factor. PLoS ONE.

[27] Kiechel M.A., Beringer L.T., Donius A.E., Komiya Y., Habas R., Wegst U.G., Schauer C.L. (2015). Osteoblast biocompatibility of premineralized, hexamethylene-1,6-diaminocarboxysulfonate cross-linked chitosan fibers. J. Biomed. Mater. Res. A.

[28] Kucharska M., Walenko K., Lewandowska-Szumiel M., Brynk T., Jaroszewicz J., Ciach T. (2015). Chitosan and composite microsphere-based scaffold for bone tissue engineering: Evaluation of tricalcium phosphate content influence on physical and biological properties. J. Mater. Sci. Mater. Med..

[29] Venkatesan J., Vinodhini P.A., Sudha P.N., Kim S.K. (2014). Chitin and chitosan composites for bone tissue regeneration. Adv. Food Nutr. Res..

[30] D’Ayala G.G., Malinconico M., Laurienzo P. (2008). Marine derived polysaccharides for biomedical applications: Chemical modification approaches. Molecules.

[31] Tan L., Hu J., Huang H., Han J., Hu H. (2015). Study of multi-functional electrospun composite nanofibrous mats for smart wound healing. Int. J. Biol. Macromol..

[32] Reddy D.H., Lee S.M. (2013). Application of magnetic chitosan composites for the removal of toxic metal and dyes from aqueous solutions. Adv. Colloid Interface Sci..

[33] Sudarshan N.R., Hoover D.G., Knorr D. (1992). Antibacterial action of chitosan. Food Biotechnol..

[34] Zheng L.Y., Zhu J.F. (2003). Study on antimicrobial activity of chitosan with different molecular weights. Carbohyd. Polym..

[35] Muzzarelli R., Tarsi R., Filippini O., Giovanetti E., Biagini G., Varaldo P.E. (1990). Antimicrobial properties of *N*-carboxybutyl chitosan. Antimicrob. Agents Chemother..

[36] Rhoades J., Roller S. (2000). Antimicrobial actions of degraded and native chitosan against spoilage organisms in laboratory media and foods. Appl. Environ. Microbiol..

[37] Younes I., Sellimi S., Rinaudo M., Jellouli K., Nasri M. (2014). Influence of acetylation degree and molecular weight of homogeneous chitosans on antibacterial and antifungal activities. Int. J. Food Microbiol..

[38] Kong M., Chen X.G., Xing K., Park H.J. (2010). Antimicrobial properties of chitosan and mode of action: A state of the art review. Int. J. Food Microbiol..

[39] Mujeeb Rahman P., Muraleedaran K., Abdul Mujeeb V.M. (2015). Applications of chitosan powder with *in situ* synthesized nano ZnO particles as an antimicrobial agent. Int. J. Biol. Macromol..

[40] Machul A., Mikolajczyk D., Regiel-Futyra A., Heczko P.B., Strus M., Arruebo M., Stochel G., Kyziol A. (2015). Study on inhibitory activity of chitosan-based materials against biofilm producing *Pseudomonas aeruginosa* strains. J. Biomater. Appl..

[41] Park S.C., Nam J.P., Kim J.H., Kim Y.M., Nah J.W., Jang M.K. (2015). Antimicrobial action of water-soluble beta-chitosan against clinical multi-drug resistant bacteria. Int. J. Mol. Sci..

[42] Fan L., Yang J., Wu H., Hu Z., Yi J., Tong J., Zhu X. (2015). Preparation and characterization of quaternary ammonium chitosan hydrogels with significant antibacterial activity. Int. J. Biol. Macromol..

[43] Li H., Peng L. (2015). Antimicrobial and antioxidant surface modification of cellulose fibers using layer-by-layer deposition of chitosan and lignosulfonates. Carbohyd. Polym..

[44] Sahariah P., Benediktssdottir B.E., Hjalmarsdottir M.A., Sigurjonsson O.E., Sorensen K.K., Thygesen M.B., Jensen K.J., Masson M. (2015). Impact of chain length on antibacterial activity and hemocompatibility of quaternary N-alkyl and n,n-dialkyl chitosan derivatives. Biomacromolecules.

[45] Sarhan W.A., Azzazy H.M. (2015). High concentration honey chitosan electrospun nanofibers: Biocompatibility and antibacterial effects. Carbohydr. Polym..

[46] Tayel A.A., Moussa S.H., Salem M.F., Mazrou K.E., El-Tras W.F. (2015). Control of citrus molds using bioactive coatings incorporated with fungal chitosan/plant extracts composite. J. Sci. Food Agric..

[47] Ben-Shalom N., Ardi R., Pinto R., Aki C., Fallik E. (2003). Controlling gray mould caused by *Botrytis cinerea* in cucumber plants by means of chitosan. Crop. Prot..

[48] Atia M.M.M., Buchenauer H., Aly A.Z., Abou-Zaid M.I. (2005). Antifungal activity of chitosan against *Phytophthora infestans* and activation of defence mechanisms in tomato to late blight. Biol. Agric. Hortic..

[49] Saharan V., Sharma G., Yadav M., Choudhary M.K., Sharma S.S., Pal A., Raliya R., Biswas P. (2015). Synthesis and *in vitro* antifungal efficacy of Cu-chitosan nanoparticles against pathogenic fungi of tomato. Int. J. Biol. Macromol..

[50] Bai R.K., Huang M.Y., Jiang Y.Y. (1988). Selective permeabilities of chitosan-acetic acid complex membrane and chitosan-polymer complex membranes for oxygen and carbon dioxide. Polym. Bull..

[51] Wang L.S., Wang C.Y., Yang C.H., Hsieh C.L., Chen S.Y., Shen C.Y., Wang J.J., Huang K.S. (2015). Synthesis and anti-fungal effect of silver nanoparticles-chitosan composite particles. Int. J. Nanomedicine.

[52] Lopez-Moya F., Colom-Valiente M.F., Martinez-Peinado P., Martinez-Lopez J.E., Puelles E., Sempere-Ortells J.M., Lopez-Llorca L.V. (2015). Carbon and nitrogen limitation increase chitosan antifungal activity in *Neurospora crassa* and fungal human pathogens. Fungal Biol..

[53] Chatterjee S., Chatterjee B.P., Guha A.K. (2014). A study on antifungal activity of water-soluble chitosan against *Macrophomina phaseolina*. Int. J. Biol. Macromol..

[54] Simonaitiene D., Brink I., Sipailiene A., Leskauskaite D. (2015). The effect of chitosan and whey proteins-chitosan films on the growth of *Penicillium expansum* in apples. J. Sci. Food Agric..

[55] Gabriel Jdos S., Tiera M.J., Tiera V.A. (2015). Synthesis, characterization, and antifungal activities of amphiphilic derivatives of diethylaminoethyl chitosan against *Aspergillus flavus*. J. Agric. Food Chem..

[56] Karagozlu M.Z., Karadeniz F., Kim S.K. (2014). Anti-HIV activities of novel synthetic peptide conjugated chitosan oligomers. Int. J. Biol. Macromol..

[57] Artan M., Karadeniz F., Karagozlu M.Z., Kim M.M., Kim S.K. (2010). *Anti*-HIV-1 activity of low molecular weight sulfated chitooligosaccharides. Carbohydr. Res..

[58] Meng J., Zhang T., Agrahari V., Ezoulin M.J., Youan B.B. (2014). Comparative biophysical properties of tenofovir-loaded, thiolated and nonthiolated chitosan nanoparticles intended for HIV prevention. Nanomedicine. (Lond.).

[59] Aghasadeghi M.R., Heidari H., Sadat S.M., Irani S., Amini S., Siadat S.D., Fazlhashemy M.E., Zabihollahi R., Atyabi S.M., Momen S.B. (2013). Lamivudine-PEGylated chitosan: A novel effective nanosized antiretroviral agent. Curr. HIV Res..

[60] Khan A.B., Thakur R.S. (2014). Formulation and evaluation of mucoadhesive microspheres of tenofovir disoproxil fumarate for intravaginal use. Curr. Drug Deliv..

[61] Ramana L.N., Sharma S., Sethuraman S., Ranga U., Krishnan U.M. (2014). Evaluation of chitosan nanoformulations as potent anti-HIV therapeutic systems. Biochim. Biophys. Acta..

[62] Belletti D., Tosi G., Forni F., Gamberini M.C., Baraldi C., Vandelli M.A., Ruozi B. (2012). Chemico-physical investigation of tenofovir loaded polymeric nanoparticles. Int. J. Pharm..

[63] Meng J., Sturgis T.F., Youan B.B. (2011). Engineering tenofovir loaded chitosan nanoparticles to maximize microbicide mucoadhesion. Eur. J. Pharm. Sci..

[64] Yang L., Chen L., Zeng R., Li C., Qiao R., Hu L., Li Z. (2010). Synthesis, nanosizing and *in vitro* drug release of a novel *anti*-HIV polymeric prodrug: Chitosan-*O*-isopropyl-5′-*O*-d4T monophosphate conjugate. Bioorg. Med. Chem..

[65] Nayak U.Y., Gopal S., Mutalik S., Ranjith A.K., Reddy M.S., Gupta P., Udupa N. (2009). Glutaraldehyde cross-linked chitosan microspheres for controlled delivery of zidovudine. J. Microencapsul..

[66] Tokoro A., Tatewaki N., Suzuki K., Mikami T., Suzuki S., Suzuki M. (1988). Growth-inhibitory effect of hexa-*N*-acetylchitohexaose and chitohexaose against Meth-A solid tumor. Chem. Pharm. Bull. (Tokyo).

[67] Lin S.Y., Chan H.Y., Shen F.H., Chen M.H., Wang Y.J., Yu C.K. (2007). Chitosan prevents the development of AOM-induced aberrant crypt foci in mice and suppressed the proliferation of AGS cells by inhibiting DNA synthesis. J. Cell Biochem..

[68] Gibot L., Chabaud S., Bouhout S., Bolduc S., Auger F.A., Moulin V.J. (2015). Anticancer properties of chitosan on human melanoma are cell line dependent. Int. J. Biol. Macromol..

[69] He B., Tao H.Y., Liu S.Q. (2014). Neuroprotective effects of carboxymethylated chitosan on hydrogen peroxide induced apoptosis in Schwann cells. Eur. J. Pharmacol..

[70] Jiang Z., Han B., Li H., Li X., Yang Y., Liu W. (2015). Preparation and anti-tumor metastasis of carboxymethyl chitosan. Carbohydr. Polym..

[71] Park J.K., Chung M.J., Choi H.N., Park Y.I. (2011). Effects of the molecular weight and the degree of deacetylation of chitosan oligosaccharides on antitumor activity. Int. J. Mol. Sci..

[72] Shen K.T., Chen M.H., Chan H.Y., Jeng J.H., Wang Y.J. (2009). Inhibitory effects of chitooligosaccharides on tumor growth and metastasis. Food Chem. Toxicol..

[73] Park P.J., Je J.Y., Kim S.K. (2003). Free radical scavenging activity of chitooligosaccharides by electron spin resonance spectrometry. J. Agric. Food Chem..

[74] Je J.Y., Park P.J., Kim S.K. (2004). Free radical scavenging properties of hetero-chitooligosaccharides using an ESR spectroscopy. Food Chem. Toxicol..

[75] Huang J., Xie H., Hu S., Xie T., Gong J., Jiang C., Ge Q., Wu Y., Liu S., Cui Y. (2015). Preparation, characterization, and biochemical activities of *N*-(2-Carboxyethyl)chitosan from squid pens. J. Agric. Food Chem..

[76] Sun T., Xie W., Xu P. (2003). Antioxidant activity of graft chitosan derivatives. Macromol. Biosci..

[77] Shukla S.K., Mishra A.K., Arotiba O.A., Mamba B.B. (2013). Chitosan-based nanomaterials: A state-of-the-art review. Int. J. Biol. Macromol..

[78] Kim I.Y., Seo S.J., Moon H.S., Yoo M.K., Park I.Y., Kim B.C., Cho C.S. (2008). Chitosan and its derivatives for tissue engineering applications. Biotechnol. Adv..

[79] Li B., Wang L., Xu F., Gang X., Demirci U., Wei D., Li Y., Feng Y., Jia D., Zhou Y. (2015). Hydrosoluble, UV-crosslinkable and injectable chitosan for patterned cell-laden microgel and rapid transdermal curing hydrogel *in vivo*. Acta. Biomater..

[80] Yang L., Wang Q., Peng L., Yue H., Zhang Z. (2015). Vascularization of repaired limb bone defects using chitosan-β-tricalcium phosphate composite as a tissue engineering bone scaffold. Mol. Med. Rep..

[81] Chameettachal S., Murab S., Vaid R., Midha S., Ghosh S. (2015). Effect of visco-elastic silk-chitosan microcomposite scaffolds on matrix deposition and biomechanical functionality for cartilage tissue engineering. J. Tissue Eng. Regen. Med..

[82] Li Q., Zhou G., Yu X., Wang T., Xi Y., Tang Z. (2015). Porous deproteinized bovine bone scaffold with three-dimensional localized drug delivery system using chitosan microspheres. Biomed. Eng. OnLine.

[83] Shapira Y., Tolmasov M., Nissan M., Reider E., Koren A., Biron T., Bitan Y., Livnat M., Ronchi G., Geuna S. (2015). Comparison of results between chitosan hollow tube and autologous nerve graft in reconstruction of peripheral nerve defect: An experimental study. Microsurgery.

[84] Tanaka N., Matsumoto I., Suzuki M., Kaneko M., Nitta K., Seguchi R., Ooi A., Takemura H. (2015). Chitosan tubes can restore the function of resected phrenic nerves. Interact. Cardiovasc. Thorac. Surg..

[85] Jian R., Yixu Y., Sheyu L., Jianhong S., Yaohua Y., Xing S., Qingfeng H., Xiaojian L., Lei Z., Yan Z. (2015). Repair of spinal cord injury by chitosan scaffold with glioma ECM and SB216763 implantation in adult rats. J. Biomed. Mater. Res. A.

[86] Correia C.O., Leite A.J., Mano J.F. (2015). Chitosan/bioactive glass nanoparticles scaffolds with shape memory properties. Carbohydr. Polym..

[87] Jabbal-Gill I., Watts P., Smith A. (2012). Chitosan-based delivery systems for mucosal vaccines. Expert Opin. Drug Deliv..

[88] Kofuji K., Qian C.J., Nishimura M., Sugiyama I., Murata Y., Kawashima S. (2005). Relationship between physicochemical characteristics and functional properties of chitosan. Eur. Polym. J..

[89] Jiang H.L., Park I.K., Shin N.R., Kang S.G., Yoo H.S., Kim S.I., Suh S.B., Akaike T., Cho C.S. (2004). *In vitro* study of the immune stimulating activity of an atrophic rhinitis vaccine associated to chitosan microspheres. Eur. J. Pharm. Biopharm..

[90] Borchard G., Lueβen H.L., de Boer A.G., Verhoef J.C., Lehr C.M., Junginger H.E. (1996). The potential of mucoadhesive polymers in enhancing intestinal peptide drug absorption. III: Effects of chitosan-glutamate and carbomer on epithelial tight junctions *in vitro*. J. Control. Release.

[91] Thanou M., Verhoef J.C., Junginger H.E. (2001). Oral drug absorption enhancement by chitosan and its derivatives. Adv. Drug Deliv. Rev..

[92] Elsabee M.Z., Morsi R.E., Al-Sabagh A.M. (2009). Surface active properties of chitosan and its derivatives. Colloids Surf. B Biointerfaces.

[93] Harada A., Kataoka K. (1995). Formation of polyion complex micelles in an aqueous milieu from a pair of oppositely-charged block copolymers with poly(ethylene glycol) segments. Macromolecules.

[94] Supper S., Anton N., Boisclair J., Seidel N., Riemenschnitter M., Curdy C., Vandamme T. (2014). Chitosan/glucose 1-phosphate as new stable *in situ* forming depot system for controlled drug delivery. Eur. J. Pharm. Biopharm..

[95] Supper S., Anton N., Seidel N., Riemenschnitter M., Curdy C., Vandamme T. (2014). Thermosensitive chitosan/glycerophosphate-based hydrogel and its derivatives in pharmaceutical and biomedical applications. Expert Opin. Drug Deliv..

[96] Lai P., Daear W., Lobenberg R., Prenner E.J. (2014). Overview of the preparation of organic polymeric nanoparticles for drug delivery based on gelatine, chitosan, poly(d,l-lactide-co-glycolic acid) and polyalkylcyanoacrylate. Colloids Surf. B Biointerfaces.

[97] Hudson D., Margaritis A. (2014). Biopolymer nanoparticle production for controlled release of biopharmaceuticals. Crit. Rev. Biotechnol..

[98] Calvo P., Remuñán-López C., Vila-Jato J.L., Alonso M.J. (1997). Novel hydrophilic chitosan-polyethylene oxide nanoparticles as protein carriers. J. Appl. Polym. Sci..

[99] Kato Y., Onishi H., Machida Y. (2005). Contribution of chitosan and its derivatives to cancer chemotherapy. In Vivo.

[100] Anraku M., Hiraga A., Iohara D., Pipkin J.D., Uekama K., Hirayama F. (2015). Slow-release of famotidine from tablets consisting of chitosan/sulfobutyl ether beta-cyclodextrin composites. Int. J. Pharm.

[101] Pereira P., Pedrosa S.S., Wymant J.M., Sayers E., Correia A., Vilanova M., Jones A.T., Gama F.M. (2015). siRNA inhibition of endocytic pathways to characterize the cellular uptake mechanisms of folate-functionalized glycol chitosan nanogels. Mol. Pharm..

[102] Kulkarni N., Wakte P., Naik J. (2015). Development of floating chitosan-xanthan beads for oral controlled release of glipizide. Int. J. Pharm. Investig..

[103] Al-Kurdi Z.I., Chowdhry B.Z., Leharne S.A., Al Omari M.M., Badwan A.A. (2015). Low molecular weight chitosan-insulin polyelectrolyte complex: Characterization and stability studies. Mar. Drugs.

[104] Gadalla H.H., Soliman G.M., Mohammed F.A., El-Sayed A.M. (2015). Development and *in vitro*/*in vivo* evaluation of Zn-pectinate microparticles reinforced with chitosan for the colonic delivery of progesterone. Drug Deliv..

[105] Jana S., Laha B., Maiti S. (2015). Boswellia gum resin/chitosan polymer composites: Controlled delivery vehicles for aceclofenac. Int. J. Biol. Macromol..

[106] Qinna N.A., Karwi Q.G., Al-Jbour N., Al-Remawi M.A., Alhussainy T.M., Al-So’ud K.A., Al Omari M.M., Badwan A.A. (2015). Influence of molecular weight and degree of deacetylation of low molecular weight chitosan on the bioactivity of oral insulin preparations. Mar. Drugs.

[107] Kim K., Ryu J.H., Lee H. (2015). Chitosan-catechol: A polymer with long-lasting mucoadhesive properties. Biomaterials.

[108] Chatterjee S., Judeh Z.M. A. (2015). Encapsulation of fish oil with *N*-stearoyl *O*-butylglyceryl chitosan using membrane and ultrasonic emulsification processes. Carbohyd. Polym..

[109] Jayasree R.S., Rathinam K., Sharma C.P. (1995). Development of artificial skin (Template) and influence of different types of sterilization procedures on wound healing pattern in rabbits and guinea pigs. J. Biomater. Appl..

[110] Ueno H., Mori T., Fujinaga T. (2001). Topical formulations and wound healing applications of chitosan. Adv. Drug Deliv. Rev..

[111] Muzzarelli R.A., Mattioli-Belmonte M., Pugnaloni A., Biagini G. (1999). Biochemistry, histology and clinical uses of chitins and chitosans in wound healing. EXS.

[112] Azuma K., Izumi R., Osaki T., Ifuku S., Morimoto M., Saimoto H., Minami S., Okamoto Y. (2015). Chitin, chitosan, and its derivatives for wound healing: Old and new materials. J. Funct. Biomater..

[113] Naseri N., Algan C., Jacobs V., John M., Oksman K., Mathew A.P. (2014). Electrospun chitosan-based nanocomposite mats reinforced with chitin nanocrystals for wound dressing. Carbohydr. Polym..

[114] Ishihara M., Obara K., Nakamura S., Fujita M., Masuoka K., Kanatani Y., Takase B., Hattori H., Morimoto Y., Ishihara M. (2006). Chitosan hydrogel as a drug delivery carrier to control angiogenesis. J. Artif. Organs.

[115] Biagini G., Bertani A., Muzzarelli R., Damadei A., DiBenedetto G., Belligolli A., Riccotti G., Zucchini C., Rizzoli C. (1991). Wound management with *N*-carboxybutyl chitosan. Biomaterials.

[116] Stone C.A., Wright H., Devaraj V.S., Clarke T., Powell R. (2000). Healing at skin graft donor sites dressed with chitosan. Br. J. Plast. Surg..

[117] Azad A.K., Sermsintham N., Chandrkrachang S., Stevens W.F. (2004). Chitosan membrane as a wound-healing dressing: Characterization and clinical application. J. Biomed. Mater. Res. B Appl. Biomater..

[118] Valentine R., Athanasiadis T., Moratti S., Hanton L., Robinson S., Wormald P.J. (2010). The efficacy of a novel chitosan gel on hemostasis and wound healing after endoscopic sinus surgery. Am. J. Rhinol. Allergy.

[119] Bennett B.L., Littlejohn L.F., Kheirabadi B.S., Butler F.K., Kotwal R.S., Dubick M.A., Bailey J.A. (2014). Management of external hemorrhage in tactical combat casualty care: Chitosan-based hemostatic gauze dressings—TCCC guidelines-change 13-05. J. Spec. Oper. Med..

[120] Hatamabadi H.R., Asayesh Zarchi F., Kariman H., Arhami Dolatabadi A., Tabatabaey A., Amini A. (2015). Celox-coated gauze for the treatment of civilian penetrating trauma: A randomized clinical trial. Trauma Mon..

[121] Nguyen N., Hasan S., Caufield L., Ling F.S., Narins C.R. (2007). Randomized controlled trial of topical hemostasis pad use for achieving vascular hemostasis following percutaneous coronary intervention. Catheter. Cardiovasc. Interv..

[122] Weng M.H. (2008). The effect of protective treatment in reducing pressure ulcers for non-invasive ventilation patients. Intensive Crit. Care Nurs..

[123] Zhang D., Zhou W., Wei B., Wang X., Tang R., Nie J., Wang J. (2015). Carboxyl-modified poly(vinyl alcohol)-cross-linked chitosan hydrogel films for potential wound dressing. Carbohydr. Polym..

[124] Jebahi S., Saoudi M., Farhat L., Oudadesse H., Rebai T., Kabir A., El Feki A., Keskes H. (2015). Effect of novel curcumin-encapsulated chitosan-bioglass drug on bone and skin repair after gamma radiation: Experimental study on a Wistar rat model. Cell Biochem. Funct..

[125] Vakili M., Rafatullah M., Salamatinia B., Abdullah A.Z., Ibrahim M.H., Tan K.B., Gholami Z., Amouzgar P. (2014). Application of chitosan and its derivatives as adsorbents for dye removal from water and wastewater: A review. Carbohyd. Polym..

[126] Boamah P.O., Huang Y., Hua M., Zhang Q., Wu J., Onumah J., Sam-Amoah L.K. (2015). Sorption of heavy metal ions onto carboxylate chitosan derivatives-A mini-review. Ecotoxicol. Environ. Saf..

[127] Tran V.S., Ngo H.H., Guo W., Zhang J., Liang S., Ton-That C., Zhang X. (2015). Typical low cost biosorbents for adsorptive removal of specific organic pollutants from water. Bioresour. Technol..

[128] Kyzas G.Z., Bikiaris D.N. (2015). Recent modifications of chitosan for adsorption applications: A critical and systematic review. Mar. Drugs.

[129] Yong S.K., Shrivastava M., Srivastava P., Kunhikrishnan A., Bolan N. (2015). Environmental applications of chitosan and its derivatives. Rev. Environ. Contam. Toxicol..

[130] Sayed S., Jardine A. (2015). Chitosan derivatives as important biorefinery intermediates. Quaternary tetraalkylammonium chitosan derivatives utilized in anion exchange chromatography for perchlorate removal. Int. J. Mol. Sci..

[131] Zhang J., Chen N., Tang Z., Yu Y., Hu Q., Feng C. (2015). A study of the mechanism of fluoride adsorption from aqueous solutions onto Fe-impregnated chitosan. Phys. Chem. Chem. Phys..

[132] Wang Y., Shi L., Gao L., Wei Q., Cui L., Hu L., Yan L., Du B. (2015). The removal of lead ions from aqueous solution by using magnetic hydroxypropyl chitosan/oxidized multiwalled carbon nanotubes composites. J. Colloid Interface Sci..

[133] Prabhu S.M., Meenakshi S. (2015). A dendrimer-like hyper branched chitosan beads toward fluoride adsorption from water. Int. J. Biol. Macromol..

[134] Li L., Li Y., Cao L., Yang C. (2015). Enhanced chromium (VI) adsorption using nanosized chitosan fibers tailored by electrospinning. Carbohyd. Polym..

[135] Kimura Y., Takahashi A., Kashiwada A., Yamada K. (2015). Removal of bisphenol derivatives through quinone oxidation by polyphenol oxidase and subsequent quinone adsorption on chitosan in the heterogeneous system. Environ. Technol..

[136] Boamah P.O., Huang Y., Hua M., Zhang Q., Liu Y., Onumah J., Wang W., Song Y. (2015). Removal of cadmium from aqueous solution using low molecular weight chitosan derivative. Carbohyd. Polym..

[137] Li H., Pan G. (2015). Simultaneous removal of harmful algal blooms and microcystins using microorganism- and chitosan-modified local Soil. Environ. Sci. Technol..

[138] Sivakami M.S., Gomathi T., Venkatesan J., Jeong H.S., Kim S.K., Sudha P.N. (2013). Preparation and characterization of nano chitosan for treatment wastewaters. Int. J. Biol. Macromol..

[139] Heber D. (2003). Herbal preparations for obesity: Are they useful?. Prim. Care.

[140] Ylitalo R., Lehtinen S., Wuolijoki E., Ylitalo P., Lehtimaki T. (2002). Cholesterol-lowering properties and safety of chitosan. Arzneimittelforschung.

[141] Gallaher D.D., Gallaher C.M., Mahrt G.J., Carr T.P., Hollingshead C.H., Hesslink R., Wise J. (2002). A glucomannan and chitosan fiber supplement decreases plasma cholesterol and increases cholesterol excretion in overweight normocholesterolemic humans. J. Am. Coll. Nutr..

[142] Maezaki Y., Tsuji K., Nakagawa Y., Kawai Y., Akimoto M., Tsugita T., Takekawa W., Terada A., Hara H., Mitsuoka T. (1993). Hypocholesterolemic effect of chitosan in adult males. Biosci. Biotechnol. Biochem..

[143] Wuolijoki E., Hirvela T., Ylitalo P. (1999). Decrease in serum LDL cholesterol with microcrystalline chitosan. Methods Find. Exp. Clin. Pharmacol..

[144] Hernández-González S.O., Manuel González-Ortiz M., Martínez-Abundis E., Robles-Cervantes J.A. (2010). Chitosan improves insulin sensitivity as determined by the euglycemic-hyperinsulinemic clamp technique in obese subjects. Nutr. Res..

[145] Pittler M.H., Abbot N.C., Harkness E.F., Ernst E. (1999). Randomized, double-blind trial of chitosan for body weight reduction. Eur. J. Clin. Nutr..

[146] Mhurchu C.N., Poppitt S.D., McGill A.T., Leahy F.E., Bennett D.A., Lin R.B., Ormrod D., Ward L., Strik C., Rodgers A. (2004). The effect of the dietary supplement, chitosan, on body weight: A randomised controlled trial in 250 overweight and obese adults. Int. J. Obes. Relat. Metab. Disord..

[147] Egras A.M., Hamilton W.R., Lenz T.L., Monaghan M.S. (2011). An evidence-based review of fat modifying supplemental weight loss products. J. Obes..

[148] Yu Y., Luo T., Liu S., Song G., Han J., Wang Y., Yao S., Feng L., Qin S. (2015). Chitosan oligosaccharides attenuate atherosclerosis and decrease non-HDL in apoE-/- mice. J. Atheroscler. Thromb..

[149] Kerch G. (2015). The potential of chitosan and its derivatives in prevention and treatment of age-related diseases. Mar. Drugs.

[150] Bento D., Staats H.F., Gonçalves T., Borges O. (2015). Development of a novel adjuvanted nasal vaccine: C48/80 associated with chitosan nanoparticles as a path to enhance mucosal immunity. Eur. J. Pharm. Biopharm..

[151] Laffleur F., Fischer A., Schmutzler M., Hintzen F., Bernkop-Schnürch A. (2015). Evaluation of functional characteristics of preactivated thiolated chitosan as potential therapeutic agent for dry mouth syndrome. Acta Biomater..

[152] Gudjónsdóttir M., Gacutan M.D., Mendes A.C., Chronakis I.S., Jespersen L., Karlsson A.H. (2015). Effects of electrospun chitosan wrapping for dry-ageing of beef, as studied by microbiological, physicochemical and low-field nuclear magnetic resonance analysis. Food Chem..

[153] Zhang X., Mysore K., Flannery E., Michel K., Severson D.W., Zhu K.Y., Duman-Scheel M. (2015). Chitosan/Interfering RNA nanoparticle mediated gene silencing in disease vector mosquito larvae. J. Vis. Exp..

